# What is Turkish women’s opinion about vaginal delivery?

**DOI:** 10.4274/tjod.59913

**Published:** 2015-06-15

**Authors:** Gülşah İlhan, Fatma Ferda Verit Atmaca, Meryem Eken, Zehra Tavukçuoğlu, Ayşegül Özel, Mucize Özdemir, Emre Sinan Güngör

**Affiliations:** 1 Süleymaniye Maternity Research and Training Hospital, Clinic of Obstetric and Gynecology, İstanbul, Turkey; 2 Zeynep Kamil Educational and Research Hospital, Clinic of Obstetric and Gynecology, İstanbul, Turkey

**Keywords:** Cesarean delivery, preferences of the route of delivery, vaginal delivery

## Abstract

**Objective:**

To determine Turkish women’s opinion about vaginal birth.

**Materials and Methods:**

This prospective cohort study was conducted in Department of Obstetrics and Gynecology of Süleymaniye Maternity Research and Training Hospital in İstanbul, Turkey, between February 2015 and April 2015. The participants of this study were 100 primiparous pregnant women who had vaginal deliveries. The women were interviewed face-to-face after the birth. Data were collected through a socio-demographic and clinical questionnaire.

**Results:**

Ninety percent of the women reported vaginal birth as the ideal mode of delivery route; a minority of the women (10%) had decided on cesarean birth before having a vaginal birth. Anxiety of pain was the major factor that influenced choice of delivery type before giving birth. After vaginal birth, 84% of women were satisfied with vaginal birth and reported that they would prefer vaginal birth for their next pregnancy. However, 16% reported that they would prefer cesarean birth for their next pregnancy due to pain of labor, pain of episiotomy, anxiety, and prolonged duration of labor.

**Conclusion:**

The results suggest the majority of women prefer to give birth vaginally and reported vaginal birth as the ideal choice.

## INTRODUCTION

Turkey has a high cesarean section birth rate. This rate has been gradually increasing in devoloping and devoloped countries. It had been reported as 37% in the Turkey Demographic and Health Survey (TDHS) 2008 report and 48% in TDHS-2013 report^(1,2,3)^. These rates are significantly higher than the acceptable rate of cesarean delivery by the World Health Organization, which is given as 15%^(4)^.

Cesarean section is life saving both for fetus and mother in the case of appropriate indications. Some studies that compared vaginal birth with cesarean section reported some advantages of cesarean section with respect to avoidance of emergency delivery, decrease in birth related fetal complications, and pelvic floor injuries^(5,6)^. Despite the improvements in prophylaxis of infection and thromboembolic events, this mode of delivery is not without risks for the mother and fetus. The increased rate of cesarean section is accompanied by abnormal placentation, uterine rupture, excessive blood loss, need for hysterectomy, injury to internal organs, increased neonatal intensive care requirement, and even maternal death. It also has considerable hindrances including prolonged hospitalization, delayed breastfeeding, higher costs and postopreative pain^(7,8)^. Besides obstetric indications, obstetrician preference and, maternal request may be reasons for cesarean section^(9,10)^.

In this study we aimed to evaluate Turkish women’s opinion about vaginal birth before and after delivery, and factors affecting this tendency.

## MATERIALS AND METHODS

This prospective cohort study was conducted in the Department of Obstetrics and Gynecology of Süleymaniye Maternity, Research and Training Hospital, İstanbul, Turkey, between February 2015 and April 2015. The participants of this study were primiparous pregnant women who gave birth vaginally. After the participants gave their informed consent, they were interviewed face-to-face within two hours of their vaginal delivery. This prospective study was approved by Süleymaniye Maternity, Research and Training Hospital’s Institutional Review Board.

A total of 100 primiparous women who had vaginal deiveries enrolled in this study. Data were collected through a socio-demographic and clinical questionnaire. These questionnaires included questions on demographics, socioeconomic status, reproductive health of patient, and also 5 open ended questions. The five open ended questions were as follows:

1. “Do you have positive or negative opinion about vaginal delivery?”,

2. “What was your preference about the ideal delivery route?” (before giving vaginal delivery),

3. “What is your preference about ideal delivery route now?” (after giving vaginal delivery),

4. “What will be your preference about next time when giving birth?”,

5. “What are your reasons?” (If the answer of 4th question was cesarean delivery).

Descriptive analysis were performed including frequency, percentage, means, and standard deviation on the demographic features, socioeconomic status and obstetric history. The answers to the first question were categorized, positive or negative opinion about vaginal delivery. He answers to the second, third, and fourth questions were coded into vaginal or cesarean delivery. The coded reponses were entered into SPSS (Statistical Package for Social Sciences) for Windows version 17.0. The answers to the fifth question were entered into Microsoft Excel exactly as the patients expressed to provide further information about preference and opinion of vaginal delivery.

## RESULTS

The mean maternal age was 25.6 years (range, 17-36 yeas). Most of the women (47%) were aged younger than 25 years. The average weeks of gestation was 39.09±2.53 weeks. All of the women were primiparous. Eight percent of women were illiterate. The education history of the patients was as follows: 32% primary school, 28% middle school, and 32% graduated from high school. Among all patients, 92% unemployed. None of them had maternal or fetal illness. All of the pregnancies were spontaneous conception. Prenatal care was considered adequate in 87% of women (Table 1).

**Figure 1:**
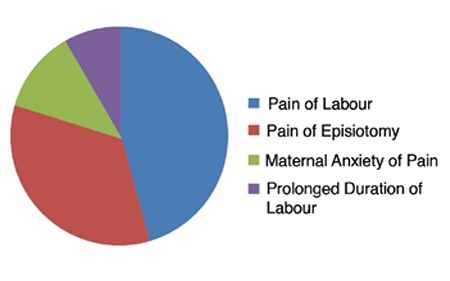
Distribution of comments after vaginal delivery

Ninety percent (n=90) of the women reported that vaginal delivery was the ideal mode of delivery, but 10% (n=10) of the women had decided on cesarean delivery before having a vaginal birth. After vaginal delivery, 84% (n=84) of the women were satisfied with vaginal delivery and had a positive opinion about vaginal birth. They also reported that they would prefer vaginal delivery for their next pregnancy. However, 16% (n=16) of the women reported that they would prefer cesarean delivery for their next pregnancy. Ten of these 16 patients had previously declared that cesarean delivery was the ideal method of giving birth. The main reason of all 10 patients (100%) was anxiety abut pain. The other less expressed reasons were anxiety about fetal injury and urinary-fecal incontinence. Although 6 of these 16 women had declared vaginal delivery as the ideal birth route, their opinion of vaginal birth had become negative after the delivery. These 16 patients who would opt for a future cesarean delivery made a total of 35 comments about their preference. The most frequent comment to fifth question was pain of labor 16/35 (45.7%) and the second frequent comment was pain of episiotomy 12/35 (34.2%). Less frequent comments were around maternal anxiety about pain 4/35 (11.4%) and prolonged duration of labour 3/35 (8.5%) (Figure 1).

## DISCUSSION

The Turkish women surveyed in this study displayed an obvious preference for vaginal delivery. Anxiety about pain was the major factor that influenced delivery type selection before giving birth. Pain of labor and episiotomy were the most commonly expressed negative concerns of Turkish women after delivery.

Preference of cesarean section are often associated with factors including maternal age, education, and socio-economic factors^(11,12,13,14,15,16,17,18,19)^. Lin et al. reported that there was a direct relationship between maternal age and demand for cesarean section^(20)^. Najmeh et al. concluded that mothers with high education were more likely to prefer cesarean section^(21)^. Rebelo et al. from Brazil and Ahmad et al. from Iran also showed similar results, but Cesaroni et al. from Italy reported that women with lower education were more willing to undergo cesarean birth^(21,22,23,24)^. A Brazilian study showed that women of higher socioeconomic status also had higher preference for cesarean section^(23)^. Fuglenes et al. in Norway and Pang et al. in Hong Kong reported that cesarean birth was preferred in 2.4% and 16.8% of primiparous women, respectively^(25,26)^. This rate was much higher in a study conducted in Iran (50.7%) by Mohammad et al. in our study, all of the women who prefered cesarean birth before delivery were pirimiparous, aged younger than 25 years, and were poorly educated^(27)^.

One of the explanations for increase in cesarean section rate is the maternal request for cesarean delivery. A meta-analysis and systematic review of Mazzoni et al. found the pooled global preference for a cesarean section to be 15.6%^(28)^. In our study, 90% (n=90) of women reported vaginal delivery was the ideal mode of delivery; 10% (n=10) had decided on cesarean delivery before having a vaginal birth. After vaginal delivery, sixteen percent (n=16) of all women reported that they would prefer a cesarean delivery for their next pregnancy.

Numerous studies concluded that the main reason for preferring cesarean was fear of pain^(25,29,30,31)^. In our study, maternal anxiety about pain was also the most commonly-expressed reservation when deciding on cesarean birth as the ideal route of delivery before giving birth. Anxiety about fetal well-being and urinary-fecal incontinence were other drawbacks expressed by the patients regarding vaginal delivery.

Our data suggest that the high cesarean birth rate in Turkey does not reflect women’s preference of delivery. However, it should be emphasized that this study is a cross-sectional study and this limits considerations regarding causality. We suggest that further studies are warranted to examine factors that influence women’s preferences in private, state, and university hospitals.

A strength of this study is that we only investigated primiparae. In this way we avoided the potential influence of previous negative birth experiences. All of the patients were interviewed within two hours of their vaginal delivery. Thus, debate over the reliability of the answers based on time arrangement was overcome.

There are some limitations of this study. The study sample was small; further studies with greater patient populations will highlight possible missing comments. This study was conducted by the doctor who delivered the patient. If the interviewer were somebody else, the comments might have been different. The interview was conducted early in the postpartum period. If the women asked the same questions after a longer interval from birth, these comments might also be different.

## Figures and Tables

**Table 1:**
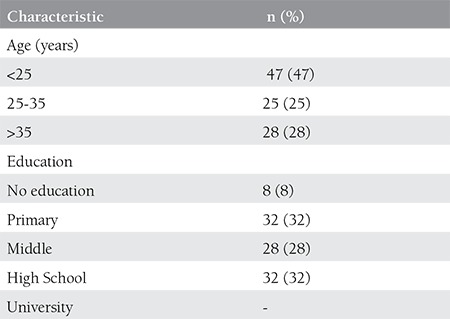
Characterics of the patients
